# Clinical warrant in medical LLM evaluation

**DOI:** 10.3389/fdgth.2026.1918724

**Published:** 2026-09-10

**Authors:** Murat Sariyar

**Affiliations:** Institute for Optimization and Data Analysis (IODA), Bern University of Applied Sciences, Bern, Switzerland

**Keywords:** clinical utility, clinical warrant, digital health, evaluation, grounding, medical large language models, retrieval-augmented generation

## Abstract

Medical large language models are evaluated through factuality, physician preference, source support, retrieval quality, safety, and clinical utility. These measures describe content and performance while leaving unresolved whether a generated claim meets the requirements for a specified clinical use. This article proposes *clinical warrant* as a use-specific judgment for a particular output–use pair. Clinical use includes reliance within a patient-related encounter, task, or care pathway, including education that can shape understanding or later action. The proposed use and the standards used to judge it are documented separately. For action-guiding outputs, clinical appropriateness is a gateway. Five further conditions examine role, case information, evidence, preserved limits, and authority/accountability. The framework asks whether these requirements are met at the point of proposed reliance, records failed or unresolved conditions and the resulting disposition, and tests whether a relevant change stops or redirects use. Fifteen constructed examples across five domains distinguish established, not established, and indeterminate warrant.

## 1 Introduction

Large language models (LLMs) made fluent medical generation plausible, while hallucination research placed dependability beyond fluency (1–3). Prior studies emphasized examination performance and factual knowledge; newer work assesses expert judgment, conversational quality, bias, harm, and user preference (4–7). Retrieval-augmented generation (RAG) links answers to sources or citations (8–10). Yet factual accuracy and source support do not establish which clinical role an output may serve.

That question concerns a particular *output–use pair*: does this generated claim meet the requirements for this role, user, and context? *Clinical warrant* is the use-specific judgment that it does. It asks whether the claim has the clinical and institutional grounds needed for reliance; legal authorization, safety, utility, and deployment decisions remain separate. Here, *clinical use* means reliance within a patient-related encounter, task, or care pathway, including education that can shape understanding or later action. An *output* is generated content; a *use claim* is an explicit or reasonably foreseeable inference or action in the declared setting; and the pair joins that claim to its specified role, user, and context. The *point of proposed reliance* is the first point at which a user or workflow can rely on the output for the declared role. Actual reliance makes the output *operative*. Action-guiding uses narrow the reasonable next steps through reassurance, delay, escalation, treatment, or medication change.

As an initial operational account, I propose five functional links. They locate different transitions from generated content to use: the assigned function (role fit), the information needed for that function (case sufficiency), the inference from evidence to claim and action threshold (evidence adequacy), the transfer of evidential limits into the output (qualifications preserved), and the passage from recommendation to an authorized, accountable decision, or action (authority/accountability). This is an analytic decomposition of failure locations, not a claim that five categories exhaust every clinical or ethical consideration. Role fit differs from authority because an assigned function does not itself confer permission to act. Evidence adequacy differs from preserved qualifications because adequate support can still be overstated when its limits disappear in generation. A defect may block several links for distinct recorded reasons, and domain-specific requirements can be nested under the relevant link.

To avoid building the conclusion into use description, the assessment uses three separate records. A *clinical-use specification* describes the target: role, user and competence, setting, available information, proposed inference or action, claimed permissions, and escalation route. It does not supply evidence or declare the use justified. A separate *assessment protocol*, fixed before output review, maps applicable clinical evidence and guidance, validated pathways, regulation, institutional policy, and scope-of-practice rules to observable requirements for the gateway and five links. Independent here means that the target declaration does not create the requirement; institutional policy counts only when issued within governing legal and professional authority. The third record documents the resulting codes and disposition. Local preference cannot convert unsupported care or authority beyond scope into warrant; unresolved applicability or conflict is coded unclear.

For action-guiding claims, clinical appropriateness is assessed separately and coded *met*, *failed*, or *unclear*; it is *not applicable* where no response, intervention, or course of care is proposed. Established warrant requires appropriateness to be met or not applicable and all five links to be met. Any failed gateway or link yields not established. An unclear gateway or link with no failure yields indeterminate. Both statuses block the use as claimed. *Disposition* records what follows: display for the specified use, repair, withholding, or authorized review. Warrant is use-specific and revisable, not a general property of the answer.

Clinical appropriateness, the closest neighboring concept, asks whether proposed care fits the circumstances and applicable standard; it may rate procedures for stated indications or individual LLM responses (11, 12). That clinical-fit judgment becomes the gateway, while warrant tests the remaining links at the point of proposed reliance. A later decision cannot retrospectively supply missing facts, restore an omitted qualification, or confer authority on the original output. Reliance after changed information, role, or authority constitutes a new pair. Appropriateness and warrant coincide only when they assess the same pair on the same grounds and authority.

Luhmann sharpened one part of this argument: the difference between representing a clinical fact and allowing it to alter what happens next. In his account, environmental events do not enter social systems as ready-made information. A system produces information when its expectations register a difference as consequential; Luhmann called this internally produced disturbance *irritation* (13, 14). Applied here, irritation contributes a design requirement rather than a clinical criterion: responsiveness to an independently established difference. Paired cases test whether a changed fact, standard, role, or permission changes status or disposition; deployment *interruptibility* asks whether the revised judgment changes actual handling. This additional step separates a responsive evaluation from a checklist that classifies an output yet leaves its use unchanged. Renal function can trigger dose review, guideline conflict can trigger adjudication, and assigned permissions can prevent execution.

## 2 Scope and conceptual approach

This paper relates medical LLM evaluation and RAG to validity, implementation, safety, and governance. Fifteen constructed output–use pairs form five triads spanning the three warrant statuses. Their purpose is explanatory; specifications and codes appear in the Supplementary Materials. The scheme and aggregation rules remain proposals for critique and domain adaptation.

## 3 Evaluation streams in recent literature

Knowledge-oriented studies assess medical questions, clinical vignettes, and expert judgment (4, 5); human evaluation adds information quality, trust, harm, and conversational usefulness (6, 7). Validity work asks whether benchmarks measure the claimed clinical ability rather than a dataset proxy (15), while reporting and governance guidance emphasizes model purpose, intended use, human oversight, and accountable deployment (16–18). Implementation research asks whether a system merits adoption through workflow fit, feasibility, and patient-level value (19–23). These streams operate at different units—answer, benchmark, system, or deployment. Warrant occupies the boundary between answer-level evidence and deployment-level action.

RAG systems are commonly evaluated for retrieval quality, citation relevance, faithfulness, answer relevance, and generation quality (9, 24). These checks are needed because citations may be incomplete, irrelevant, or poorly matched to the claims they appear to support (10). Clinical use adds a further demand: the retrieved material must be direct enough for the proposed inference. A guideline may support a general statement while leaving a patient-specific recommendation, triage decision, or medication change underdetermined (Table 1).

**Table 1:** Adjacent evaluation streams, with a direct comparison of clinical appropriateness and proposed clinical warrant.

Evaluation stream	Representative focus	What the assessment establishes	Relation to clinical warrant
Knowledge and expert evaluation	Accuracy, reasoning, preference, harm, trust	Whether answers satisfy content-level criteria	Whether an answer satisfying those criteria may serve this role in this case
RAG and source evaluation	Retrieval, citation relevance, faithfulness, answer support	Whether answer content is linked to evidence	Whether the evidence is direct enough for the implied clinical conclusion
Validity and reporting	Construct validity, documentation, intended use	Whether the evaluation target is specified	Whether this output matches the intended use at the moment of action
Implementation and utility	Workflow fit, feasibility, patient-level value	Whether a tool affects care processes or outcomes	Warrant is pair-level and does not establish beneficial deployment effects
Safety	Potential harm and risk control	Whether expected harms are acceptably controlled	Warrant does not determine whether residual risk is acceptable
Intended use and governance	Scope, permissions, oversight, accountability	What the system is designed or permitted to do and who remains accountable	Whether this output has the case information, evidence, and qualifications required for that permitted use
Clinical appropriateness	Clinical option for an indication	Whether the proposed response or care fits the stated circumstances and applicable standard	Serves as the clinical-fit gateway for action-guiding warrant; coded not applicable when no care option is proposed
Proposed clinical warrant	Generated claim entering a declared use	Whether the target satisfies independent clinical-fit and five-link requirements at the specified point	Uses the appropriateness gateway and adds role fit, case sufficiency, evidence adequacy, preserved qualifications, authority/accountability, and disposition

## 4 Deriving warrant from adjacent assessments

Adjacent traditions supply the substantive grounds for the five links. Intended-use reporting motivates role fit (17, 18); RAND/UCLA indication methods motivate case sufficiency (11); evidence-based medicine and GRADE motivate evidence adequacy and preserved qualifications (25–27); omission research shows how generation can erase a limit (28); and governance contributes permissions and accountability (18, 29). Clinical warrant organizes these standards around a particular use. It keeps the proposed use, reference requirements, and resulting judgment distinct; makes the output–use pair the unit of judgment; anchors assessment to the point of proposed reliance; separates condition codes from status and disposition; and tests whether a changed requirement redirects use. The question is no longer whether an answer is good in general, but whether independently grounded requirements support this particular use.

Safety and clinical utility answer different questions. *Safety* concerns possible harms and their mitigation; *clinical utility* concerns comparative effects on decisions, workflow, outcomes, or resources. Warrant asks whether this output is justified for the specified use under the stated role, case, evidence, and authority. A low-risk recommendation may exceed its evidence or role, while a high-risk emergency alert may have warrant under an adequate threshold and escalation pathway. Utility aggregates effects across a deployment; warrant assesses each output–use pair. A beneficial deployment can contain unwarranted outputs, and a warranted output can occur within an ineffective deployment.

Appropriateness supplies the clinical fit, and governance defines system scope, oversight, permissions, and accountability. Both may also be used to audit individual outputs and incidents. Warrant then asks, for a particular output, whether the required case information, evidence, qualifications, and authority support the proposed use. The record identifies the relevant grounds, every failed or unresolved requirement, and what should happen to the output. An authorized system may still produce an unwarranted flag when required variables are absent or a review prompt becomes autonomous execution.

Paired cases provide a counterfactual check on this distinction. Holding the patient and proposed care constant while changing only the output’s role isolates whether authorization and accountability alter warrant while clinical appropriateness remains stable. Holding the role constant while changing renal function, urgency, or the applicable pathway tests sensitivity to a decision-relevant clinical fact. Warrant should change only when a declared, decision-relevant condition changes; the applicable reference standard determines the expected result.

A user’s professional or non-professional status does not determine warrant. Warrant depends on the assigned task, required competence, available information, degree of autonomy, decision authority, and escalation path. A clinician may hold prescribing authority while the available inputs remain insufficient for a particular dose. An automated workflow may process complete inputs while exceeding its assigned permissions. The framework therefore applies to patients, professionals, and automated workflows (21).

A clinical-use specification is complete when it describes the proposed reliance without deciding its acceptability. Defensibility belongs to the separate assessment protocol: each requirement must be traceable to applicable evidence or guidance, validated clinical pathways, regulation, institutional policy adopted within governing authority, or scope-of-practice rules. The protocol records source, applicability, precedence, and observable criterion before output review. This separation exposes both the proposed use and the standard used to judge it to scrutiny. A silent or conflicting standard yields an indeterminate result and referral to the person or body authorized to resolve it (29).

Evidence adequacy expresses the relation between a source and the proposed use. The evidence cited or relied on must fit the population, setting, intervention or exposure, outcome, and action threshold, with attention to certainty, currency, and conflicts. A source can support a background proposition while remaining too indirect for a patient-specific action. More specific or consequential actions usually require more direct evidence; a validated urgent pathway can justify escalation at a lower evidential threshold than diagnosis or treatment.

The relevance of context depends on the proposed use. Urgency can change the information needed and the threshold for action. A patient’s goals may rule out an otherwise reasonable recommendation; unavailable local services may make a proposed next step impossible. Equity concerns arise when evidence excludes relevant groups, subgroup limits are omitted, or access and authority are distributed unfairly (30).

## 5 Illustrative examples and failure mechanisms

Radiologists identified inappropriate ChatGPT advice about scheduling mammography after COVID-19 vaccination (31). The example contains two distinct inferences: from vaccination to possible transient adenopathy, and from that association to what this patient should do tomorrow. Evidence supports the first while leaving the second underdetermined. Symptoms, cancer risk, prior imaging, local policy, and scheduling authority determine whether delay is appropriate. Repair therefore preserves the educational explanation, removes the unsupported scheduling instruction, and refers the decision to the imaging pathway. Table 2 uses this and four further cases to show how case completeness, evidence directness, and authority alter the permitted use.

**Table 2:** Clinical warrant in two matched output–use contrasts and one indeterminate case.

Scenario and use claim	Warrant status	Gateway and condition profile	Disposition
*Mammography—education:* Patient asks why vaccination can affect images. Output explains transient lymph-node enlargement and leaves scheduling to the imaging pathway	**Established**	*Appropriateness—N/A:* no care option proposed. *Role—met:* education. *Case—met:* general explanation needs no scheduling facts. *Evidence—met:* mechanism supported. *Qualifications—met:* facility requirements preserved. *Authority—met:* imaging pathway schedules	*Display for education.* Follow facility instructions
*Mammography—scheduling:* Patient asks whether to cancel tomorrow. Output advises delay based only on recent vaccination	*Not established*	*Appropriateness—failed:* cancelation unsupported. *Role—met:* scheduling advice. *Case—failed:* symptoms, risk, prior imaging, and policy unavailable. *Evidence—failed:* vaccination alone is insufficient. *Qualifications—failed:* exceptions omitted. *Authority—failed:* model assumes scheduling control	*Repair.* Remove delay advice; refer to the imaging pathway
*Medication support—review:* System recommends a rule-supported temporary hold, displays the complete case, evidence, and alternatives, and requests prescriber approval	*Established*	*Appropriateness—met:* hold fits the stated case. *Role—met:* decision support. *Case—met:* required facts available. *Evidence—met:* approved rule applies. *Qualifications—met:* alternatives retained. *Authority—met:* prescriber decides	*Display for review;* log decision
*Medication support—execution:* With the same facts and proposed hold, the system executes before prescriber approval	*Not established*	*Appropriateness—met:* same clinical option. *Role—failed:* execution exceeds support role. *Case—met. Evidence—met. Qualifications—met. Authority—failed:* prescriber approval bypassed	*Withhold execution;* route for approval
*Guideline conflict—adjudication.* Output gives a provisional dose under one of two conflicting guidelines, discloses the conflict, and requests adjudication; precedence is unspecified	*Indeterminate*	*Appropriateness—unclear:* applicable dose unresolved. *Role—met:* provisional advice. *Case—met:* required facts available. *Evidence—unclear:* guideline precedence unresolved. *Qualifications—met:* conflict disclosed. *Authority—met:* prescriber decides	*Escalate* for guideline or institutional adjudication

Within each contrast, the medical background remains stable while the proposed use changes.

The same output–use distinction applies when algorithmic outputs enter clinical workflows. Validation of the Epic Sepsis Model concerned discrimination and calibration. Warrant for using its score to trigger an alert additionally depends on who receives the alert, what action follows, and how false and missed alerts are handled (32). Autonomous screening and surgical support raise analogous questions (30, 33–35). Clinical Pharmacogenetics Implementation Consortium (CPIC) guidance likewise separates using an available genotype from deciding whether to test; a valid drug–gene statement provides evidence but does not determine indication, dose, alternatives, urgency, or prescribing authority (36).

Generation can change the force of the underlying evidence. A fluent summary may overgeneralize findings, omit limiting attributes, or sound more decisive than the literature permits (37–39). Patient-friendly simplification can improve readability while deleting clinically relevant meaning (40). Omission-focused work shows how excluded facts alter simulated diagnostic reasoning (28). Qualifiers, exclusions, uncertainty, subgroup restrictions, and escalation conditions often determine whether an answer may become advice. Cautious wording can still accompany a directive recommendation for delay, medication change, or reassurance. Evaluators therefore assess the use claim directly and treat a disclaimer as one qualification among several.

Some cases retain factual accuracy, source support, and cautious wording while the permitted use changes. The same proposition can support education while action requires further case information, evidence, or authority. The same clinical option can retain appropriateness while role or authority changes warrant. The *blocker profile* shows every failed or unresolved condition; a primary repair target is named only when one correction is a prerequisite for the others.

## 6 Operational evaluation

A future evaluation could test the framework in six prespecified steps. First, before inspecting sampled outputs, the study would document a descriptive clinical-use specification and an assessment protocol based on applicable sources. The protocol would state applicable evidence, pathways, policy hierarchy, permissions, and observable requirements; unresolved standards or authority would be coded unclear. Second, the study would identify explicit and foreseeable use claims. A foreseeable claim would need to follow from the output, interface, and declared workflow without adding a new clinical premise and be available to the specified user as an inference or action. Separate claims would be assessed individually and combined only when they support the same proposed use. Third, the study would assess clinical appropriateness separately for action-guiding claims and then assess all five links. Evidence standards would depend on the use: topic relevance might support education, whereas treatment or triage would require direct evidence and the applicable threshold or pathway.

Fourth, code every link as met, failed, or unclear and retain the complete blocker profile. Record a primary repair target only when resolving it is a prerequisite to the remaining repairs; report independent blocks jointly. Established requires a met or not-applicable gateway and five met links. Any failure yields not established; any unresolved gateway or link with no failure yields indeterminate. Fifth, report status separately from *disposition*: repair, withhold, or escalate a blocked claim. Display means only that warrant does not block the specified use; safety, utility, regulation, governance, and deployment decisions remain separate. Sixth, cross-tabulate warrant with factuality, source support, safety, and utility while reporting the appropriateness gateway. Reports should identify target specifications, reference sources and criteria, condition codes, blocker profiles, status rates, dispositions, and paired-case responses.

The medication contrast holds the case and clinically appropriate option constant. Warrant changes because review and autonomous execution assign different roles and authority. The example therefore isolates what appropriateness alone cannot decide.

Requirements would vary by domain. A patient-portal study would distinguish education from self-management and define escalation; medication support would require the indication, dose, relevant comorbidities, interactions, and prescribing authority; triage would depend on applicable red flags and emergency thresholds. In a RAG-based study, assessment would follow retrieval and evidence selection because the generated answer is what enters use (41, 42). Before sampling, domain experts would translate all five links into observable requirements and could add prespecified subconditions. Any study that splits, merges, reorders, or removes links would be evaluating a modified framework and should report the change explicitly.

An evaluation set could implement these controlled contrasts. Each pair would state what changes, what remains fixed, and whether warrant is expected to remain stable or change. Evaluation could then report sensitivity to specified blockers and undesired sensitivity to clinically irrelevant changes. This would test whether the assessment distinguishes the five requirements and assigns status for the stated reason (43–45).

Automation could identify use claims, cited sources, omitted qualifiers, unsupported claims, and shifts from uncertainty to directive language. Expert reviewers would retain the judgment of evidence adequacy, authority, and local standards. Each judgment should identify the guideline, evidence standard, clinical pathway, institutional policy, or accountable professional judgment on which it rests (29).

## 7 Discussion

Condition-level findings guide the remedy. Structured intake addresses case sufficiency; evidence or reference-standard review addresses support and contested thresholds; generation and interface changes preserve qualifications; system permissions and escalation routes govern authority. The recorded failure should identify the control capable of correcting it. An aggregate status remains useful, while condition codes show the standards and policies supporting the judgment.

The proposal is most useful when clinical fit and system authorization appear acceptable, yet the particular output lacks the facts, evidence, qualifications, or authority required for reliance. Appropriateness should agree with warrant on clinical fit, and governance on permissions and accountability. Comparative evaluation can identify the responsible condition. If the same judgment is always reached, the framework functions chiefly as a shared reporting structure. Because target description and reference requirements are stored separately, agreement cannot be produced merely by redefining the intended use after seeing an output.

The original judgment remains attached to its specified pair. Changed patient data, evidence, thresholds, permissions, or service availability create an updated pair for continued use and require reassessment. Paired cases test the new judgment; deployment audits test whether it stops or redirects use. This requires update triggers and accountable review rather than one-time certification. Specifications and judgments remain open to correction without rewriting the earlier assessment.

## 8 Limitations

The triads explain the three statuses; estimates of frequency and practical effect require empirical studies. The five links are provisional analytic failure locations rather than a closed taxonomy. Domain studies may test whether they remain distinguishable, require subdivision, or omit a recurrent blocker. Warrant also depends on a defensible assessment protocol: conflicting guidance, unresolved measurement validity, disputed authority, or undefined thresholds yield indeterminate status. Separating target and standard makes their relation auditable but does not resolve legitimate disagreement among standards. Future comparisons should determine whether the proposal identifies causes of blocked use more precisely or primarily provides a shared reporting structure.

## 9 Conclusion

Clinical warrant asks whether a generated claim meets independently grounded requirements for a specified clinical use. It complements content support and clinical appropriateness by asking whether the required case information accompanies the proposed use, the output preserves the qualifications on which that use depends, and reliance remains within assigned authority. It is a use-specific judgment, not legal authorization or a final deployment decision. Its practical test is whether a decision-relevant change alters the judgment and stops or redirects use before the claim becomes operative.
