# Supplementary material for: Clinical warrant in medical LLM evaluation
Source: Front Digit Health. 2026 Sep 10;8:1918724. doi: 10.3389/fdgth.2026.1918724 (PMC13601723; doi:10.3389/fdgth.2026.1918724)
Supplement: Supplementary file 1 [file Datasheet1.pdf]

Supplementary Material 1: Proposed Clinical Warrant Example Coding Rules

Purpose

These rules operationalize one proposed version of the framework for the 15 constructed cases. They are an initial scheme for critique, comparative evaluation, and domain adaptation. Clinical appropriateness is assessed as a gateway, followed by all five functional links. Domain teams translate independently applicable clinical and institutional standards into observable requirements and may add prespecified subconditions. A study that changes the gateway or five-link structure would be evaluating a modified framework and should report the change.

The cases are arranged in five domain triads. Each triad contains one established profile, one with a resolved failure, and one with an unresolved requirement but no failure. The matrix records how gateway and link codes lead to status, a complete blocker profile, and disposition. The author specifies the assumptions and adjacent-signal codes for illustration.

Unit of analysis

One specified output–use pair is the unit of analysis. Clinical use means reliance within a patient-related encounter, task, or care pathway, including education that can shape understanding or later action. The output is generated content; a use claim is an explicit or reasonably foreseeable inference or action in the declared setting. A foreseeable claim must follow from the output, interface, and declared workflow without adding a new clinical premise, and it must be available to the specified user as an inference or action. Separate claims are assessed individually; claims are combined only when they jointly support the same proposed use. The point of proposed reliance is the first point at which a user or workflow can rely on the output for the declared role.

The assessment uses three separate records:

1. The **clinical-use specification** describes the target: role and decision context, user and competence, setting, information available at the point of proposed reliance, proposed inference or action, claimed permissions, prohibited actions, and escalation route. It neither supplies evidence nor declares the use justified.
2. Before output review, the **assessment protocol** maps applicable clinical evidence and guidance, validated pathways, regulation, institutional policy, and scope-of-practice rules to observable requirements for the appropriateness gateway and five links. Independent means that the target declaration does not create the requirement; institutional policy qualifies only when issued within governing legal and professional authority. The protocol records each source, its applicability and precedence, and the required evidence for a code. Local preference cannot make unsupported care or authority beyond scope warranted.
3. The **assessment record** contains gateway and link codes, blocker profile, status, and disposition.

The same experts may contribute to these records, but target description and normative judgment remain separately documented and open to challenge. The matrix's `governing_reference_standard` field summarizes the independent standard used in each constructed case.

Derivation and relation to adjacent assessments

Adjacent approaches supply the grounds for the five links. Intended-use reporting and governance motivate `role_fit` and `authority_accountable`; indication-based appropriateness methods motivate `case_sufficient`; evidence-based medicine and GRADE motivate `evidence_adequate` and `qualifications_preserved`; omission research identifies the risk that generation removes a decision-relevant limit. Clinical warrant organizes these standards around a specified pair. It separates target description, reference requirements, and judgment; anchors assessment to the point of proposed reliance; distinguishes condition codes from status and disposition; and tests responses to changed requirements.

Clinical appropriateness asks whether a proposed response, intervention, or course of care fits the stated circumstances and applicable standard. It is assessed separately and coded `met`, `failed`, or `unclear` for action-guiding claims and `not_applicable` where no care option is proposed. This clinical-fit judgment serves as the gateway to warrant; the five links then test the remaining requirements for reliance.

The judgments coincide only when they assess the same pair on the same grounds and authority. The medication contrast holds the case and clinically appropriate option constant while changing review into autonomous execution; appropriateness remains met while warrant changes through role and authority. Education uses `not_applicable`. Later information or authority belongs to an updated pair and does not retrospectively alter the original judgment.

Governance may itself audit individual outputs and incidents. Within that broader process, warrant supplies the output-level judgment needed when a generated claim is about to enter use.

Condition codes

After the gateway, the scheme assesses five analytically distinct transitions from output to use:

1. assigned function: `role_fit`;
2. information required for that function: `case_sufficient`;
3. inference from evidence to claim and any action threshold: `evidence_adequate`;
4. transfer of evidential limits into the generated output: `qualifications_preserved`;
5. passage from recommendation to authorized and accountable decision or action: `authority_accountable`.

These are proposed failure locations rather than an exhaustive list of clinical or ethical values. Domain-specific requirements are nested under one or more links. Each link is coded `met`, `failed`, or `unclear`.

- `met` means that the condition is satisfied under the independently sourced assessment protocol and information available at the point of proposed reliance.
- `failed` means that, under a resolved reference standard, the specified output–use pair violates the condition. Proceeding despite the known absence of information required by that standard is a failure.
- `unclear` records an unresolved governing standard, applicability judgment, measurement validity question, allocation of authority, or ethical or institutional approval. It suspends the warrant judgment for that use claim pending adjudication.

1. `role_fit`: The use claim remains within the educational, review, advisory, triage, or action function declared before output review. It concerns what the output is assigned to do.
2. `case_sufficient`: Information required by the prespecified pathway for that role and use is available.
3. `evidence_adequate`: The cited evidence or rule is applicable and sufficiently direct to carry the use claim and any action threshold.
4. `qualifications_preserved`: The output keeps decision-relevant uncertainty, exclusions, alternatives, and preference-sensitive limits visible.
5. `authority_accountable`: The person or workflow may decide or act as claimed, and responsibility or escalation is documented.

Role and authority can fail together for different reasons: the output assumes an undeclared function, and the actor or workflow lacks permission to execute it. Evidence and qualifications can likewise fail together when support is too weak and the output also suppresses its limits. Each reason remains separately recorded.

Contextual factors are assigned to the condition they affect:

- urgency and time sensitivity: `case_sufficient` and, when they change the action threshold, `evidence_adequate`;
- patient goals and preferences: `case_sufficient` and `qualifications_preserved`;
- resource availability: `case_sufficient` for the declared setting;
- equity: `evidence_adequate`, `qualifications_preserved`, or `authority_accountable`, depending on whether the issue concerns evidence, omitted limits, or access and authority.

Warrant status

The gateway and all five links are aggregated conjunctively:

- `established`: appropriateness is `met` or `not_applicable`, and all five links are `met`.
- `not_established`: the gateway or at least one link is `failed`.
- `indeterminate`: the gateway or at least one link is `unclear`, and none is `failed`.

Warrant is use-specific and revisable. Both `not_established` and `indeterminate` block the use as presently claimed. A demonstrated failure calls for repair or withholding; an unresolved criterion calls for accountable adjudication. `Established` means that the requirements for the specified use are satisfied. It does not amount to legal authorization, proof of safety or utility, or a final deployment decision.

Disposition

Status records the judgment; disposition records what should happen next.

- `display`: neither gateway nor link blocks display for the specified use. Separate safety, utility, governance, and deployment decisions still apply.
- `repair`: revise wording or remove a blocked use claim under the existing target and protocol. A changed role, proposed action, claimed permission, or escalation route requires a new clinical-use specification. A changed normative requirement or threshold requires a separately justified revision of the assessment protocol before reevaluation.
- `withhold`: block display or execution of an unrepaired failed use claim.
- `escalate`: refer the unresolved standard or case to the person or team authorized and accountable for the decision.

Blocker profile

The matrix records every `failed` or `unclear` gateway or link in `blocked_conditions`; an unblocked profile records `none`. No universal ordering is imposed. A study may designate a primary repair target only when correcting one condition is a prerequisite for addressing the others. Independent blocks remain jointly reported.

Paired-case check

A controlled pair states what changes, what remains fixed, and the expected response. A missed interruption leaves judgment unchanged after a prespecified blocker changes; a spurious interruption changes it after an irrelevant difference. The original judgment remains attached to its pair. Changed facts, evidence, thresholds, role, or authority create an updated pair for continued use. Deployment interruptibility asks whether the updated judgment stops or redirects live use.

Adjacent evaluation signals

In actual model evaluation, the appropriateness gateway is reported separately, and warrant is cross-tabulated with safety and clinical utility. Safety, utility, regulation, governance, and deployment review can still block a pair whose warrant status is established. The constructed matrix also codes the narrower adjacent signals below.

- `factuality_pass = yes`: the medical background proposition expressed by the output is accurate within the information supplied. Case applicability and support for the use claim are coded separately.
- `source_support_pass = yes`: an identifiable source or rule supports the expressed background proposition. Evidence adequacy separately assesses population, setting, intervention, outcome, and action-threshold fit.
- `caution_or_escalation_language_present = yes`: the output contains caution, uncertainty, a disclaimer, or escalation language. This code describes wording; safety and any directive use claim receive their own evaluation.
- `no`: the corresponding signal is contradicted or absent.
- `unclear`: the information supplied leaves the signal unresolved.

The adjacent signals are descriptive measures. In the constructed matrix, at least one example in every warrant-status class is factually accurate, source-supported, and cautiously worded. Their presence across all classes shows why output–use status requires the five warrant conditions.
